# 4-Hydrazinopyridinium chloride

**DOI:** 10.1107/S1600536809026348

**Published:** 2009-07-11

**Authors:** René T. Boeré, Mohammad R. Hassan

**Affiliations:** a Department of Chemistry and Biochemistry, The University of Lethbridge, Lethbridge, Alberta, Canada T1K3M4

## Abstract

In the title compound, C_5_H_8_N_3_^+^·Cl^−^, the cation and the anion lie on a mirror plane and are hydrogen bonded in a three-dimensional network *via* the H atoms of the two hydrazine N atoms. The pyridine N atom is protonated and hydrogen bonded to the terminal hydrazine N atom.

## Related literature

For related structures, see: Lima *et al.* (2008 ▶); Hammerl *et al.* (2001 ▶). For background to the synthesis, see: Mann *et al.* (1959 ▶).
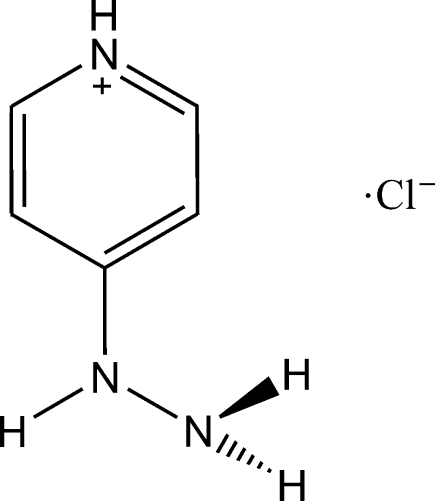


## Experimental

### 

#### Crystal data

C_5_H_8_N_3_^+^·Cl^−^
*M* *_r_* = 145.59
Monoclinic, 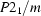

*a* = 6.9526 (11) Å
*b* = 6.434 (1) Å
*c* = 7.7432 (12) Å
β = 95.316 (1)°
*V* = 344.89 (9) Å^3^
*Z* = 2
Mo *K*α radiation
μ = 0.46 mm^−1^
*T* = 173 K
0.27 × 0.19 × 0.18 mm

#### Data collection

Bruker APEXII CCD area-detector diffractometer
Absorption correction: multi-scan (*SADABS*; Bruker, 2006 ▶) *T*_min_ = 0.884, *T*_max_ = 0.920
4968 measured reflections
855 independent reflections
840 reflections with *I* > 2σ(*I*)
*R*_int_ = 0.016

#### Refinement

*R*[*F* ^2^ > 2σ(*F* ^2^)] = 0.022
*wR*(*F* ^2^) = 0.060
*S* = 1.13
855 reflections
63 parameters
H atoms treated by a mixture of independent and constrained refinement
Δρ_max_ = 0.32 e Å^−3^
Δρ_min_ = −0.20 e Å^−3^

### 

Data collection: *APEX2* (Bruker, 2006 ▶); cell refinement: *SAINT-Plus* (Bruker, 2006 ▶); data reduction: *SAINT-Plus*; program(s) used to solve structure: *SHELXD* (Sheldrick, 2008 ▶); program(s) used to refine structure: *SHELXTL* (Sheldrick, 2008 ▶); molecular graphics: *Mercury* (Macrae *et al.*, 2006 ▶); software used to prepare material for publication: *publCIF* (Westrip, 2009 ▶).

## Supplementary Material

Crystal structure: contains datablocks I, global. DOI: 10.1107/S1600536809026348/pv2178sup1.cif

Structure factors: contains datablocks I. DOI: 10.1107/S1600536809026348/pv2178Isup2.hkl

Additional supplementary materials:  crystallographic information; 3D view; checkCIF report

## Figures and Tables

**Table 1:** Hydrogen-bond geometry (Å, °)

*D*—H⋯*A*	*D*—H	H⋯*A*	*D*⋯*A*	*D*—H⋯*A*
N7—H7⋯Cl1	0.89 (2)	2.25 (2)	3.1358 (14)	176.7 (19)
N8—H8⋯Cl1^i^	0.849 (14)	2.905 (14)	3.1970 (14)	102.4 (11)
N1—H1⋯N8^ii^	0.89 (2)	1.92 (2)	2.8069 (19)	172.0 (19)

Symmetry codes: (i) 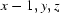; (ii) 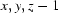.

**Fig. 1.**
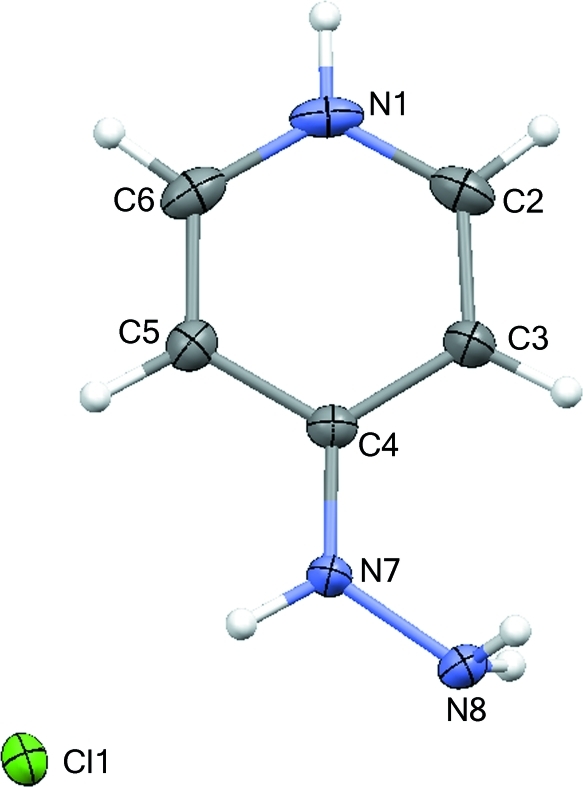
A view of (I) plotted with displacement ellipsoids at 50% probability level.

**Fig. 2.**
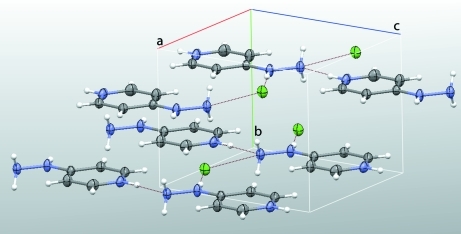
Packing diagram of (I) showing the network of H-bonds.

## supplementary crystallographic information

### Comment

In the structure of the title compound, (I), (Figure 1.) both ions crystallize on the mirror plane perpendicular to *b* with a separation of *b*/2 (3.217 Å). In consequence, the N7, N8 and N7—H atom are coplanar with the aromatic ring, and thus the out-of-plane H atoms on N8 are in a staggered conformation with respect to the N7—H atom. The local conformation of the aryl-hydrazine is similar to that observed in only two known crystal structures, both of phenylhydrazine, namely [(C_6_H_5_NHNH_2_)H]_2_(N_3_),(II), Hammerl *et al.* (2001) and [C_6_H_5_NHNH_3_]Cl, (III), Lima *et al.* (2008). The former contains both PhNHNH_2_ and PhNHNH_3_^+^ in the lattice. However, in (I) it is the more basic pyridine N1 that is protonated, but which also forms a strong H bond to the terminal hydrazinyl N8 (*D*···*A* = 2.8069 (19) Å). This bond is comparable in strength to the linking H bond between PhNHNH_2_ and PhNHNH_3_^+^ in (II). The structures of (II) and (III) are also composed of essentially flat sheets of Aryl—N units, with inter-planar separations of 3.497 and 3.378 Å, respectively.

There are additional H bonds between the N7—H and the N8—H atoms and the chloride anion which, in conjunction with the infinite chains of N1—H to N8 bonds, result in the formation of planar hydrogen-bonded sheets (Figure 2), with N···Cl distances very comparable to those found in (III).

In summary, the structure of (I) has a higher symmetry than (II) and (III) and is tightly packed due to a network of strong H bonds.

### Experimental

4-Chloropyridine (1.1 mmol, 4.20 g) and pure hydrazine hydrate (1.1 mmol, 1.63 g) were added to 10 ml of 1-propanol. After refluxing for 48 h, the mixture was cooled to 273 K and washed with cold 1-propanol. Recrystallization from methanol yielded 3.6 g of the title compound (I) as colorless needles in 65% yield. The compound (I) has a melting point of 516–517 K, which was in agreement with published values (Mann *et al.* 1959).

### Refinement

Space group determination was ambiguous between *P*2_1_ and *P*2_1_/*m* because of poor E-statistics. However, the structure was successfully solved using the SHELXD procedure (Sheldrick, 2008) and refined in *P*2_1_/*m*. The origin of the ambiguous E-statistics became obvious after structure solution, as every atom except for the two N8 hydrogen atoms are found on a crystallographic mirror plane. All H atoms were located in a difference map. N-bound H atoms were freely refined with the constraint *U*_iso_(H) = 1.2*U*_eq_(N). The C-bound H atoms were placed in calculated positions (C—H = 0.95 Å) and refined as riding with *U*_iso_(H) = 1.2*U*_eq_(C).

### Crystal data

**Table:**

C_5_H_8_N_3_^+^·Cl^−^	*F*(000) = 152
*M**_r_* = 145.59	*D*_x_ = 1.402 Mg m^−^^3^
Monoclinic, *P*2_1_/*m*	Mo *K*α radiation, λ = 0.71073 Å
Hall symbol: -P 2yb	Cell parameters from 4500 reflections
*a* = 6.9526 (11) Å	θ = 2.6–27.5°
*b* = 6.434 (1) Å	µ = 0.46 mm^−^^1^
*c* = 7.7432 (12) Å	*T* = 173 K
β = 95.316 (1)°	Block, colourless
*V* = 344.89 (9) Å^3^	0.27 × 0.19 × 0.18 mm
*Z* = 2	

### Data collection

**Table:**

Bruker APEXII CCD area-detector diffractometer	855 independent reflections
Radiation source: fine-focus sealed tube	840 reflections with *I* > 2σ(*I*)
graphite	*R*_int_ = 0.016
φ and ω scans	θ_max_ = 27.5°, θ_min_ = 2.6°
Absorption correction: multi-scan (*SADABS*; Bruker, 2006)	*h* = −9→9
*T*_min_ = 0.884, *T*_max_ = 0.920	*k* = −8→8
4968 measured reflections	*l* = −10→10

### Refinement

**Table:**

Refinement on *F*^2^	Secondary atom site location: notdet
Least-squares matrix: full	Hydrogen site location: difference Fourier map
*R*[*F*^2^ > 2σ(*F*^2^)] = 0.022	H atoms treated by a mixture of independent and constrained refinement
*wR*(*F*^2^) = 0.060	*w* = 1/[σ^2^(*F*_o_^2^) + (0.0256*P*)^2^ + 0.1361*P*] where *P* = (*F*_o_^2^ + 2*F*_c_^2^)/3
*S* = 1.13	(Δ/σ)_max_ < 0.001
855 reflections	Δρ_max_ = 0.32 e Å^−^^3^
63 parameters	Δρ_min_ = −0.20 e Å^−^^3^
0 restraints	Extinction correction: *SHELXTL* (Sheldrick, 2008), Fc^*^=kFc[1+0.001xFc^2^λ^3^/sin(2θ)]^-1/4^
Primary atom site location: dual	Extinction coefficient: 0.038 (6)

### Special details

**Table:**

Geometry. All e.s.d.'s (except the e.s.d. in the dihedral angle between two l.s. planes) are estimated using the full covariance matrix. The cell e.s.d.'s are taken into account individually in the estimation of e.s.d.'s in distances, angles and torsion angles; correlations between e.s.d.'s in cell parameters are only used when they are defined by crystal symmetry. An approximate (isotropic) treatment of cell e.s.d.'s is used for estimating e.s.d.'s involving l.s. planes.
Refinement. Refinement of *F*^2^ against ALL reflections. The weighted *R*-factor *wR* and goodness of fit *S* are based on *F*^2^, conventional *R*-factors *R* are based on *F*, with *F* set to zero for negative *F*^2^. The threshold expression of *F*^2^ > σ(*F*^2^) is used only for calculating *R*-factors(gt) *etc*. and is not relevant to the choice of reflections for refinement. *R*-factors based on *F*^2^ are statistically about twice as large as those based on *F*, and *R*- factors based on ALL data will be even larger.

### Fractional atomic coordinates and isotropic or equivalent isotropic displacement parameters (Å^2^)

**Table:**

	*x*	*y*	*z*	*U*_iso_*/*U*_eq_	
Cl1	0.83751 (5)	0.2500	0.59397 (4)	0.02334 (14)	
N1	0.3602 (2)	0.2500	−0.14229 (17)	0.0266 (3)	
N7	0.42703 (18)	0.2500	0.38856 (16)	0.0210 (3)	
N8	0.27431 (18)	0.2500	0.49629 (16)	0.0212 (3)	
C2	0.2020 (3)	0.2500	−0.0545 (2)	0.0274 (3)	
H2	0.0779	0.2500	−0.1172	0.033*	
C3	0.2159 (2)	0.2500	0.12280 (19)	0.0238 (3)	
H3	0.1027	0.2500	0.1827	0.029*	
C4	0.4010 (2)	0.2500	0.21599 (18)	0.0183 (3)	
C5	0.5645 (2)	0.2500	0.11846 (19)	0.0213 (3)	
H5	0.6912	0.2500	0.1762	0.026*	
C6	0.5387 (3)	0.2500	−0.0580 (2)	0.0253 (3)	
H6	0.6483	0.2500	−0.1227	0.030*	
H1	0.345 (3)	0.2500	−0.257 (3)	0.030*	
H7	0.545 (3)	0.2500	0.443 (3)	0.030*	
H8	0.209 (2)	0.139 (2)	0.4769 (18)	0.030*	

### Atomic displacement parameters (Å^2^)

**Table:**

	*U*^11^	*U*^22^	*U*^33^	*U*^12^	*U*^13^	*U*^23^
Cl1	0.01569 (19)	0.0324 (2)	0.0216 (2)	0.000	−0.00012 (12)	0.000
N1	0.0429 (8)	0.0248 (7)	0.0120 (6)	0.000	0.0026 (5)	0.000
N7	0.0150 (6)	0.0346 (7)	0.0133 (5)	0.000	0.0005 (4)	0.000
N8	0.0191 (6)	0.0297 (7)	0.0151 (6)	0.000	0.0041 (5)	0.000
C2	0.0305 (8)	0.0311 (8)	0.0191 (7)	0.000	−0.0058 (6)	0.000
C3	0.0201 (7)	0.0335 (8)	0.0174 (7)	0.000	−0.0003 (5)	0.000
C4	0.0197 (7)	0.0202 (7)	0.0150 (6)	0.000	0.0012 (5)	0.000
C5	0.0206 (7)	0.0228 (7)	0.0209 (7)	0.000	0.0044 (5)	0.000
C6	0.0345 (8)	0.0216 (7)	0.0215 (7)	0.000	0.0114 (6)	0.000

### Geometric parameters (Å, °)

**Table:**

N1—C2	1.345 (2)	C2—H2	0.9500
N1—C6	1.348 (2)	C3—C4	1.416 (2)
N1—H1	0.89 (2)	C3—H3	0.9500
N7—C4	1.3317 (18)	C4—C5	1.422 (2)
N7—N8	1.4097 (17)	C5—C6	1.361 (2)
N7—H7	0.89 (2)	C5—H5	0.9500
N8—H8	0.849 (14)	C6—H6	0.9500
C2—C3	1.368 (2)		
			
C2—N1—C6	120.97 (13)	C2—C3—H3	120.4
C2—N1—H1	118.7 (13)	C4—C3—H3	120.4
C6—N1—H1	120.3 (13)	N7—C4—C3	122.96 (14)
C4—N7—N8	123.64 (12)	N7—C4—C5	119.47 (13)
C4—N7—H7	120.4 (13)	C3—C4—C5	117.58 (13)
N8—N7—H7	115.9 (13)	C6—C5—C4	119.71 (15)
N7—N8—H8	108.3 (10)	C6—C5—H5	120.1
N1—C2—C3	121.48 (15)	C4—C5—H5	120.1
N1—C2—H2	119.3	N1—C6—C5	121.05 (15)
C3—C2—H2	119.3	N1—C6—H6	119.5
C2—C3—C4	119.21 (15)	C5—C6—H6	119.5
			
C6—N1—C2—C3	0.0	C2—C3—C4—C5	0.0
N1—C2—C3—C4	0.0	N7—C4—C5—C6	180.0
N8—N7—C4—C3	0.0	C3—C4—C5—C6	0.0
N8—N7—C4—C5	180.0	C2—N1—C6—C5	0.0
C2—C3—C4—N7	180.0	C4—C5—C6—N1	0.0

### Hydrogen-bond geometry (Å, °)

**Table:**

*D*—H···*A*	*D*—H	H···*A*	*D*···*A*	*D*—H···*A*
N7—H7···Cl1	0.89 (2)	2.25 (2)	3.1358 (14)	177 (2)
N8—H8···Cl1^i^	0.849 (14)	2.905 (14)	3.1970 (14)	102.4 (11)
N1—H1···N8^ii^	0.89 (2)	1.92 (2)	2.8069 (19)	172 (2)

Symmetry codes: (i) *x*−1, *y*, *z*; (ii) *x*, *y*, *z*−1.
